# Exploitation of Engineered Light-Switchable Myosin XI for Nanotechnological Applications

**DOI:** 10.1021/acsnano.3c05137

**Published:** 2023-08-28

**Authors:** Aseem Salhotra, Mohammad A Rahman, Paul V Ruijgrok, Christoph R Meinecke, Marko Ušaj, Sasha Zemsky, Frida W Lindberg, Pradheebha Surendiran, Roman W. Lyttleton, Heiner Linke, Till Korten, Zev Bryant, Alf Månsson

**Affiliations:** † Department of Chemistry and Biomedical Sciences, Linnaeus University, 39182 Kalmar, Sweden; ‡ Department of Bioengineering, Stanford University, 94305 Stanford, California, United States; § NanoLundLund University, Box 118, 22100 Lund, Sweden; ∥ Solid State Physics, Lund University, Box 118, 22100 Lund, Sweden; ⊥ Center for Microtechnologies, Technische Universität Chemnitz, 09126 Chemnitz, Germany; # B CUBE - Center for Molecular Bioengineering and Physics of Life, Technische Universität Dresden, D-01307 Dresden, Germany

**Keywords:** engineered myosin XI, actin, light-switchable motor, nanofabrication, surface chemistry, spatiotemporal motility control

## Abstract

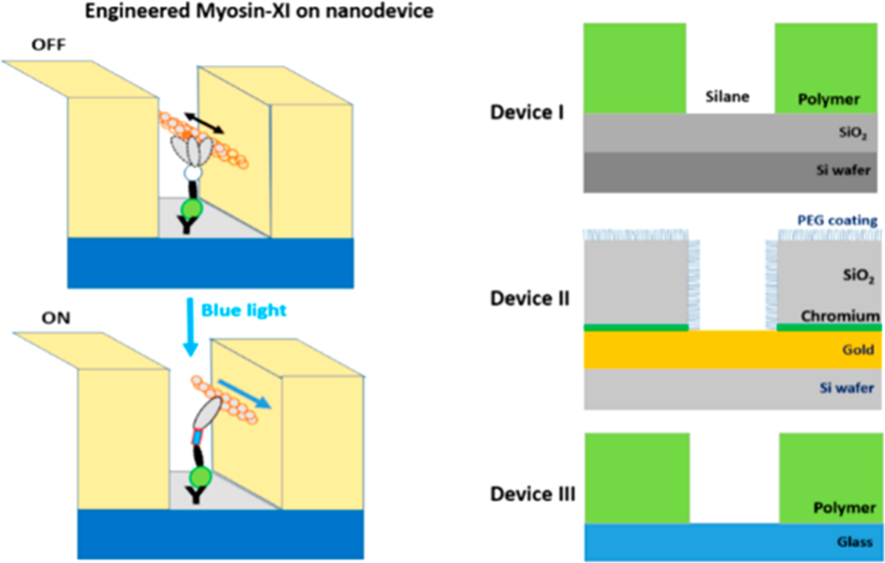

For certain nanotechnological applications of the contractile proteins actin and myosin, *e.g*., in biosensing and network-based biocomputation, it would be desirable to temporarily switch on/off motile function in parts of nanostructured devices, *e.g*., for sorting or programming. Myosin XI motor constructs, engineered with a light-switchable domain for switching actin motility between high and low velocities (light-sensitive motors (LSMs) below), are promising in this regard. However, they were not designed for use in nanotechnology, where longevity of operation, long shelf life, and selectivity of function in specific regions of a nanofabricated network are important. Here, we tested if these criteria can be fulfilled using existing LSM constructs or if additional developments will be required. We demonstrated extended shelf life as well as longevity of the actin-propelling function compared to those in previous studies. We also evaluated several approaches for selective immobilization with a maintained actin propelling function in dedicated nanochannels only. Whereas selectivity was feasible using certain nanopatterning combinations, the reproducibility was not satisfactory. In summary, the study demonstrates the feasibility of using engineered light-controlled myosin XI motors for myosin-driven actin transport in nanotechnological applications. Before use for, *e.g*., sorting or programming, additional work is however needed to achieve reproducibility of the nanofabrication and, further, optimize the motor properties.

Molecular motors like myosin, kinesin, and dynein play key roles in cells through conversion of chemical energy into mechanical work by coupling the turnover of adenosine triphosphate (ATP) with mechanical action on cytoskeletal filaments (actin filaments and microtubules). This mechanochemical function has been exploited in nanotechnology following the finding that isolated myosin motor fragments can propel actin filaments when immobilized on flat surfaces in the *in vitro* motility assay.^1,2^ This assay has been very useful in functional studies of different myosin motors. A path toward its use in nanotechnological applications was laid when it was found that myosin motor-driven transportation of actin filaments could be limited to microsized tracks.^3,4^ Subsequently, the actin-myosin system has been tested for use in several applications (reviewed in^5−10^), most notably analyte detection with ultrafast biosensing^11^ and network-based biocomputation.^12−15^ In the latter application, myosin motors are immobilized in nanochannels to distribute actin filaments along appropriately designed nanofabricated networks to solve mathematical problems encoded in the networks.

Previous studies^16−18^ describe the requirements for designing nanotracks for effective guiding of actin filament transport. Actin sliding velocity can be controlled using genetically modified myosin motors,^19^ myosin inhibitors,^20^ or varying experimental conditions,^21,22^ but these approaches are not suitable for localized spatiotemporal control of actin transport. One possibility to achieve such localized control may be to use the thermoresponsive polymer poly(*N*-isopropylacrylamide) (PNIPAM), controlled by localized heating.^23,24^ However, although PNIPAM is useful to switch on/off microtubule transport, it has not yet been applied to highly localized on/off switching as required in biocomputation or sorting applications in biosensing. Neither has the method been adapted for actin and myosin where unpredictable challenges may appear, considering a number of differences from the kinesin-1-microtubule motor systems (*cf*.^9^; see also^25,26^).

We here propose another way to perform localized control of actin filament transport by substituting wild-type myosin II motor fragments, used previously in nanodevices,^11,12,17,27−31^ with engineered myosin motors that can be locally switched on and off by altered illumination.^32,33^ In the following, we denote these motors as light-sensitive motors (LSMs). Without blue light, LSM-powered actin filaments move quite slowly. In contrast, when the blue light (470–490 nm in the current study) is turned “on” the actin propulsion rate increases within seconds.^32,33^ The use of LSM for spatiotemporal control in nanodevices has easily recognizable advantages compared to polymer (PNIPAM) grafting mentioned above, as there is no need for localized surface engineering for polymer grafting or thermal control. However, there are other potential problems, and we here investigate the potential to overcome these. An issue that we expected to be particularly challenging was to achieve LSM-induced actin motility selectively in nanoscale channels but not in surrounding areas. Thus, the actin-myosin nanodevices that have been used so far, with excellent selectivity but without potential for spatiotemporal control, have been designed in a lengthy process.^17,18,34,35^ In these devices, the myosin II motor fragment heavy meromyosin (HMM) adsorbs in a motility-supporting form in trimethylchlorosilane derivatized nanochannels surrounded by motility-suppressing oxygen-plasma-treated polymer resists. The basis for the selectivity is a disordered, hydrophobic C-terminal domain of HMM that preferentially adsorbs to hydrophobic surfaces and positively charged actin binding regions that preferentially adsorb to negatively charged surfaces such as oxygen plasma treated polymer resists.^36,37^ The chemical properties of the C-terminal of the LSMs are entirely different, with the protein in the standard expression mode being fused to yellow fluorescent protein (YFP) (externally nearly identical to green fluorescent protein, GFP). Accordingly, in studies using standard nonpatterned *in vitro* motility assays, the LSMs have thus far been surface-immobilized via anti-GFP antibodies which, in turn, have been adsorbed to nitrocellulose-coated glass surfaces. These surfaces cannot be nanostructured for use in nanodevices. This requirement for nanostructuring was what initially prompted the tests of varied silanized surfaces with respect to their capacity to support motility using myosin II-based HMM.^28,35^

Nevertheless, for control of transport in nanonetworks, driven by LSM, it is essential to achieve selective actin-propelling myosin function in the nanochannels but no motility on surrounding areas.^12,16−18,38^ By systematic investigations, we find, just as initially believed, that this is highly challenging to achieve reproducibly. Importantly, however, we found that it is feasible. As other important prerequisites for the use of LSMs in nanodevices, we also report increased storage shelf life and operational longevity of the engineered motors. In summary, our studies support the feasibility of our main aim of using light-sensitive myosin motors in actomyosin-driven nanodevices. However, it will be critical to improve the reproducibility of nanofabrication to fine-tune all surface properties for selective motor immobilization on predetermined areas only.

## Results and Discussion

### General

**Figure 1:**
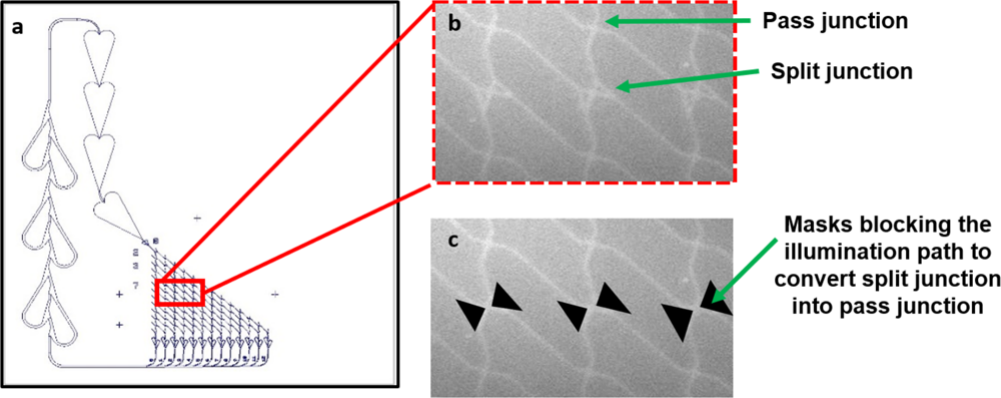
Proposed concept of programming a biocomputation network using light-switchable motors with illumination masks at selected sites. (a). Schematic of a biocomputation network. (b) Zoomed image projection of network area (∼28 × 40 μm^2^) showing nanochannels observed with Alexa-647-ADP labeling of HMM myosin II motor fragments by locking the fluorescent nucleotide in the active site by vanadate treatment. (c) Same image of the network as in (b), illustrating schematically how illumination can be selectively blocked using microfabricated barriers located in conjugate field plane of the illumination path. This will allow biocomputation junctions in selected sites to transform from one type to another, consistent with programming of the network to solve another computational problem. An idea for practical implementation of blocking, as suggested in panel (c), is given in Figure S1.

**Figure 2:**
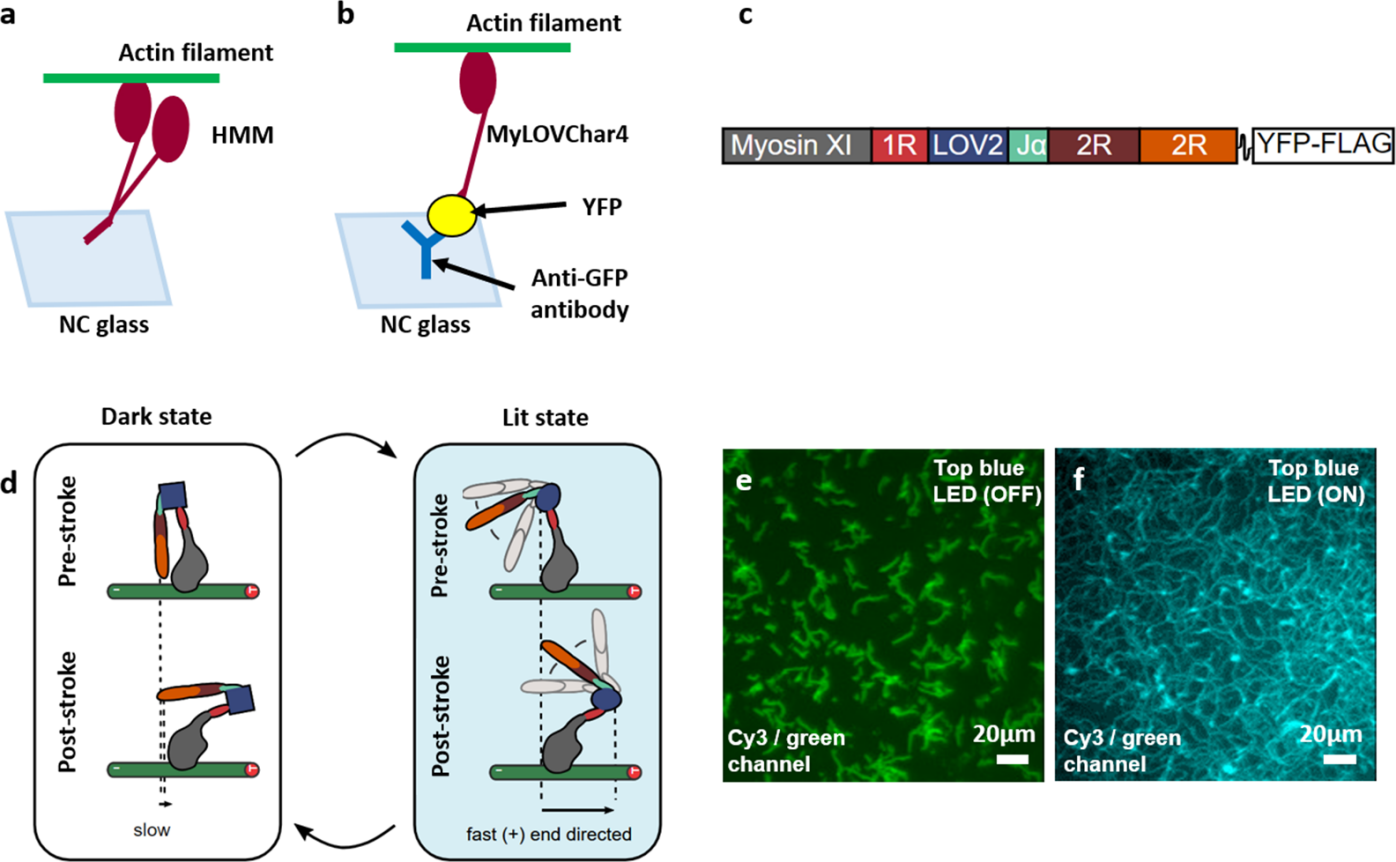
Surface immobilization of light-switchable myosin (MyLOVChar4) compared to myosin II motor fragment and switching mechanism and change in function upon blue light illumination. (a) Adsorption on nitrocellulose glass (NC-glass) surface in case of HMM (motor fragment of myosin II). (b) Light-switchable myosin motor (MyLOVChar4) is fused to yellow fluorescent protein (YFP) which binds to anti-green fluorescent protein (anti-GFP) antibody, in turn adsorbed on the nitrocellulose-coated glass surface. (c) Molecular diagram of the light -switchable motor MyLOVChar4.^33^ The construct fuses the catalytic motor domain from a fast plant myosin XI (gray) to a lever arm with a hairpin structure formed by an LOV2 domain (blue) flanked by spectrin repeats (red, brown, and orange) and contains a C-terminal yellow fluorescent protein (YFP) and FLAG tag. (d) Schematic of the optical switching mechanism of MyLOVChar4. In the dark state (left box), MyLOVChar4 produces a small stroke (black arrow), illustrated by comparing the (actin-projected) position of the tip of the lever arm between prestroke conformation (top panel) and post stroke conformation (bottom panel). In the lit state (right box), the last rigid residue of the LOV2 domain becomes the effective end of the lever arm, resulting in a larger stroke of the motor (black arrow below dotted lines). A larger stroke of the motor results in a larger velocity of the propelled filaments. The fractional population of motors in an ensemble can be controlled from mostly in the dark state to mostly in the lit state by the presence of blue light. Figure adapted with permission from Nature Chemical Biology.^33^ Copyright, the authors (P.V. Ruijgrok et al.) under exclusive license to Springer Nature America Inc. (e) Image stack (20 frames, maximum projection) showing restricted myosin propelled actin filament motility under green light illumination only (Cy3 filter set, motors switched OFF). (f) Image stack (20 frames, maximum projection) showing myosin propelled actin filament motility (extended actin filament paths) with the addition of blue light illumination (motors switched ON). Actin filaments labeled with Rhodamine phalloidin, ON/OFF switching performed using blue LED illuminator (see Materials and Methods). Images in (e, f) pseudocolored to indicate type of illumination.

For the purpose of switching motility ON and OFF by altered illumination, *e.g*., for programming of a biocomputation network (Figure 1) or for sorting actin filaments in biosensing applications, we propose using engineered LSM fragments in combination with nanofabricated networks. The latter are produced using material combinations that have not been previously tested for this purpose. The LSM fragments consist of an engineered myosin XI construct with a domain organization different from that of HMM from myosin II (Figure 2a–c). The monomeric myosin XI construct MyLOVChar4 has an artificial lever arm consisting of a blue light-sensitive light-oxygen-sensing (LOV2) domain, flanked by spectrin repeats, and a C-terminal yellow fluorescent protein (YFP).^32,33^ This design allows switching via a light-induced change in the flexibility of the LOV2 domain, which leads to altered displacement of the distal part of the myosin lever arm during the motor cycle (Figure 2d). The visualization of fluorescent myosin motor fragments in the nanochannels in Figure 1b suggests that optical resolution should be sufficient to distinguish different parts of the nanochannels to allow fully selective blue-light switching where different parts are ON and OFF simultaneously, as indicated schematically in Figure 1c. While not implemented here, we expect to achieve it straightforwardly, as described in Figure S1.

The switching behavior of LSMs on artificial surfaces has been observed earlier in the *in vitro* motility assay (IVMA), where motors were immobilized via adsorbed antibodies on nitrocellulose surfaces.^32,33^ In contrast to HMM (proteolytic motor fragment of myosin II) that is adsorbed in functional form directly onto the functionalized surfaces, LSMs are generally immobilized to surfaces via their fused YFP moiety, which interacts with surface-adsorbed, anti-GFP antibodies (Figure 2a,b). The light-sensitive motors change their action under the influence of light switching, causing actin filaments to move at different speeds (and possibly direction) if blue light (*e.g*., using fluorescein isothiocyanate (FITC) filter-set in epi-fluorescence microscope or dedicated illuminator) is switched ON or OFF. For the motor construct MyLOVChar4^33^ that we use here, this leads to fast sliding velocity when blue light is ON and more restricted and slow movements if the blue light is OFF (Figure 2e,f).

**Figure 3:**
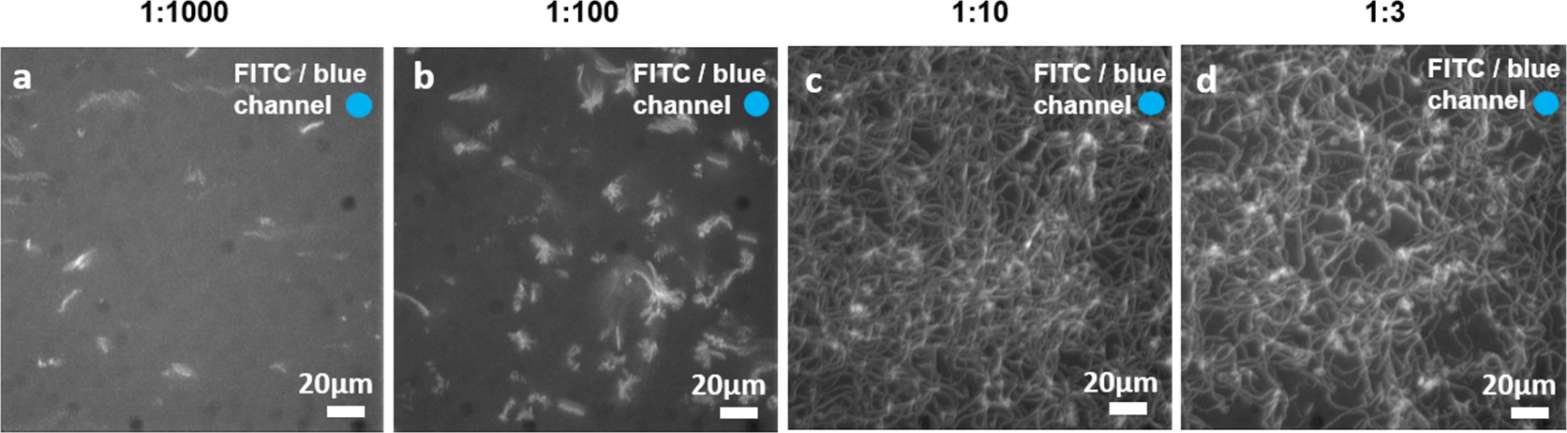
Motility of actin filaments produced by LSM after surface incubation with different anti-GFP antibody concentrations before incubation with LSM at 150 nM. (a) Image stack (50 frames, maximum projection) showing very limited motility under 1000-times dilution of antibody. (b) Image stack as in (a), showing no motility under 100-times dilution of antibody. (c) Image stack (100 frames, maximum projection) showing motility with uniform distribution under 10-times dilution of antibody. (d) Image stack as in (c), showing motility with less uniform distribution under 3-times dilution of antibody. Experiments were performed using nitrocellulose surfaces after antibody incubation for 2 min (Temperature, 25 °C). Actin filaments labeled with Alexa-488 phalloidin illuminated using fluorescein isothiocyanate (FITC) filter set of microscope (i.e., LSM motility condition always ON). Blue circle in top right corner indicates color of illuminating light.

The surface density of immobilized LSMs depends on the distribution of anti-GFP antibodies on the surface. Before performing other experiments, we optimized the antibody surface density for good motor function on nitrocellulose surfaces. To that end, the antibody stock solution (catalog No. MAB3580; from Millipore) was diluted in different ratios of 1:1000, 1:100, 1:10, and 1:3 in phosphate-buffered saline (PBS) solution (1X) supplemented with bovine serum albumin (BSA) (1 mg/mL). In the cases of 1000 and 100 times diluted antibodies, no motility was observed (Figure 3a,b), but both 10- and three-times diluted antibodies resulted in motility, with better function obtained in the case of 10 times compared to 3 times dilution (Figure 3c,d). Based on these findings, 10 times diluted antibody was utilized as the standard concentration for all further experiments.

**Figure 4:**
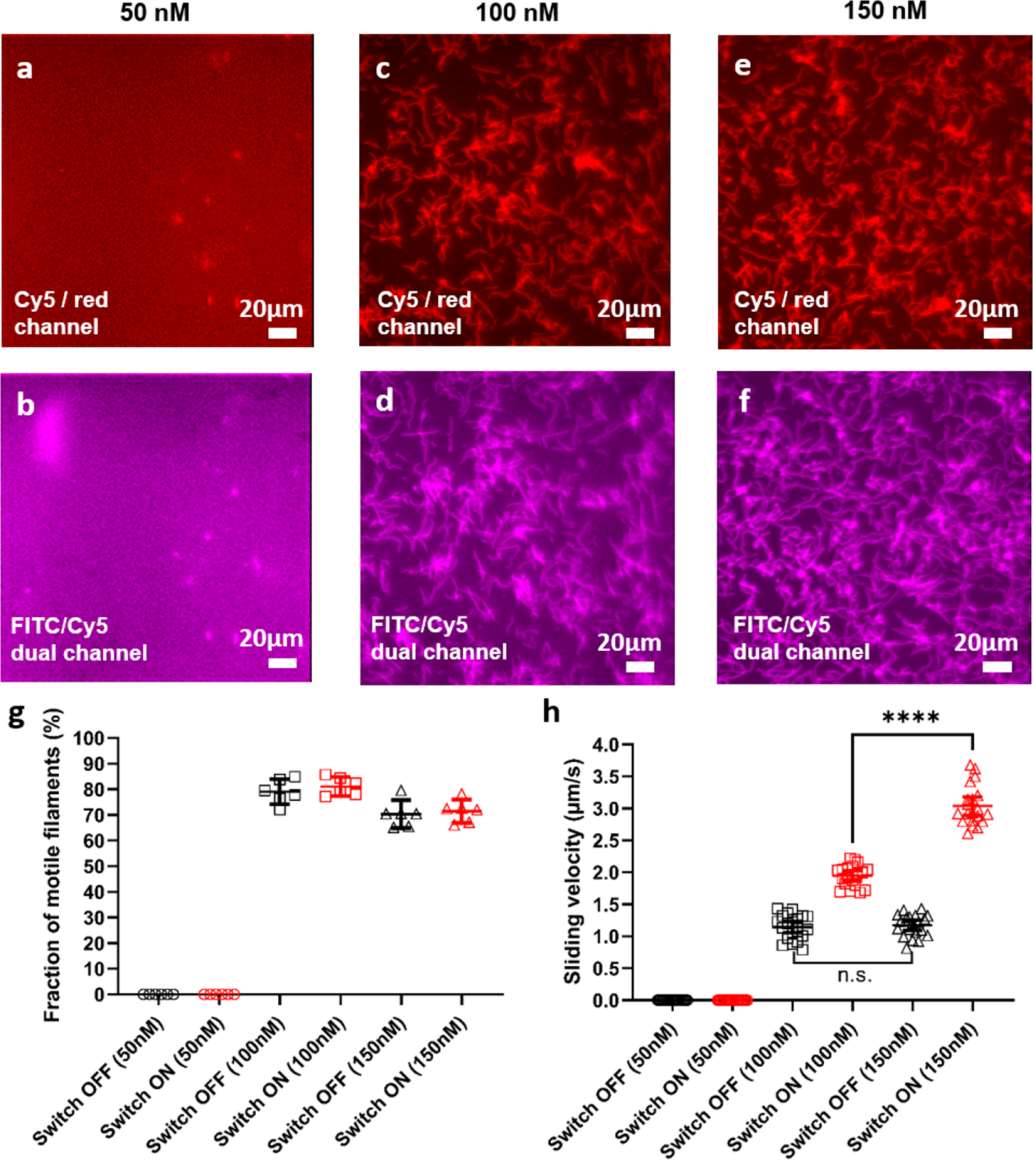
Motile function of actin filaments propelled by LSM after incubation with the motors at different concentrations. (a, b) Image stacks (100 frames, maximum projection contrast and brightness adjusted) showing minimal distribution and no motility for 50 nM motor concentration under Cy5 filter (LSM switched OFF) and FITC/Cy5 dual filter (switched ON), respectively. (c, d) Image stacks (20 frames), showing somewhat nonuniform distribution of motility for 100 nM motor concentration under Cy5 filter (OFF) and FITC/Cy5 dual filter (ON), respectively. (e, f) Image stacks as in (c, d) (20 frames) showing more uniform distribution of motility for 150 nM motor concentration under Cy5 filter (OFF) and FITC/Cy5 dual filter (ON), respectively. (g) Measured fraction of motile filaments at motor concentration 50, 100, and 150 nM. Note similar fraction of motile filaments in this experiment under “ON” and “OFF” conditions. (h) Measured sliding velocity at motor concentration 50, 100, and 150 nM. **** Statistically significant difference (*p* < 0.0001; *t* test); n.s. no significant difference (*p* ≈ 0.652). Note, highest velocity contrast between “ON” and “OFF” conditions at 150 nM. In (g, h), for fraction, six different flow cell regions of interest (three on each experimental occasion) and for velocity, 20 different filaments (10 for each experimental occasion) were analyzed for each condition distributed over two different experimental occasions (two different days). All filaments were pooled in the statistical analysis as further motivated in the Materials and Methods. Data shown as mean ±95% confidence intervals superimposed on data for individual filaments. Temperature, 24.5–25.5 °C. Actin filaments labeled with Alexa-647 phalloidin. Switching performed using illumination (indicated by pseudocoloring of images) through Cy5 filter set (OFF; red color code) and FITC/Cy5 dual filter set (ON; purple color code). Images in each column are from a given field of view before and after ON switching.

With the above standard dilution of anti-GFP antibodies, further tests were performed to find a suitable motor concentration that gives both uniform actin filament binding distribution, high fraction of motile filaments, and smooth and high sliding velocity. First, using nitrocellulose surfaces, three different LSM incubation concentrations, 50, 100, and 150 nM, were compared and checked with blue light switching between on and off. Motility was observed for both 100 and 150 nM but not for the 50 nM motor concentration (Figure 4a–f). Uniformity for actin binding and function was best with a 150 nM motor concentration (Figure 4c–f). The fraction of motile filaments was similar for 100 and 150 nM whether blue light was ON or OFF. The sliding velocity was higher for the 150 nM than the 100 nM motor concentration (*p* < 0.0001) with blue light ON. Furthermore, also the velocity contrast between blue light ON and OFF conditions was highest for the 150 nM incubation concentration (Figure 4h). This follows from the higher velocity under ON conditions at the higher LSM incubation conditions but similar velocity under OFF conditions (*p* ≈ 0.652). Thus, 150 nM motor concentration was kept as standard for all experiments below and is used unless otherwise stated.

### Longevity of operation and shelf life

**Figure 5:**
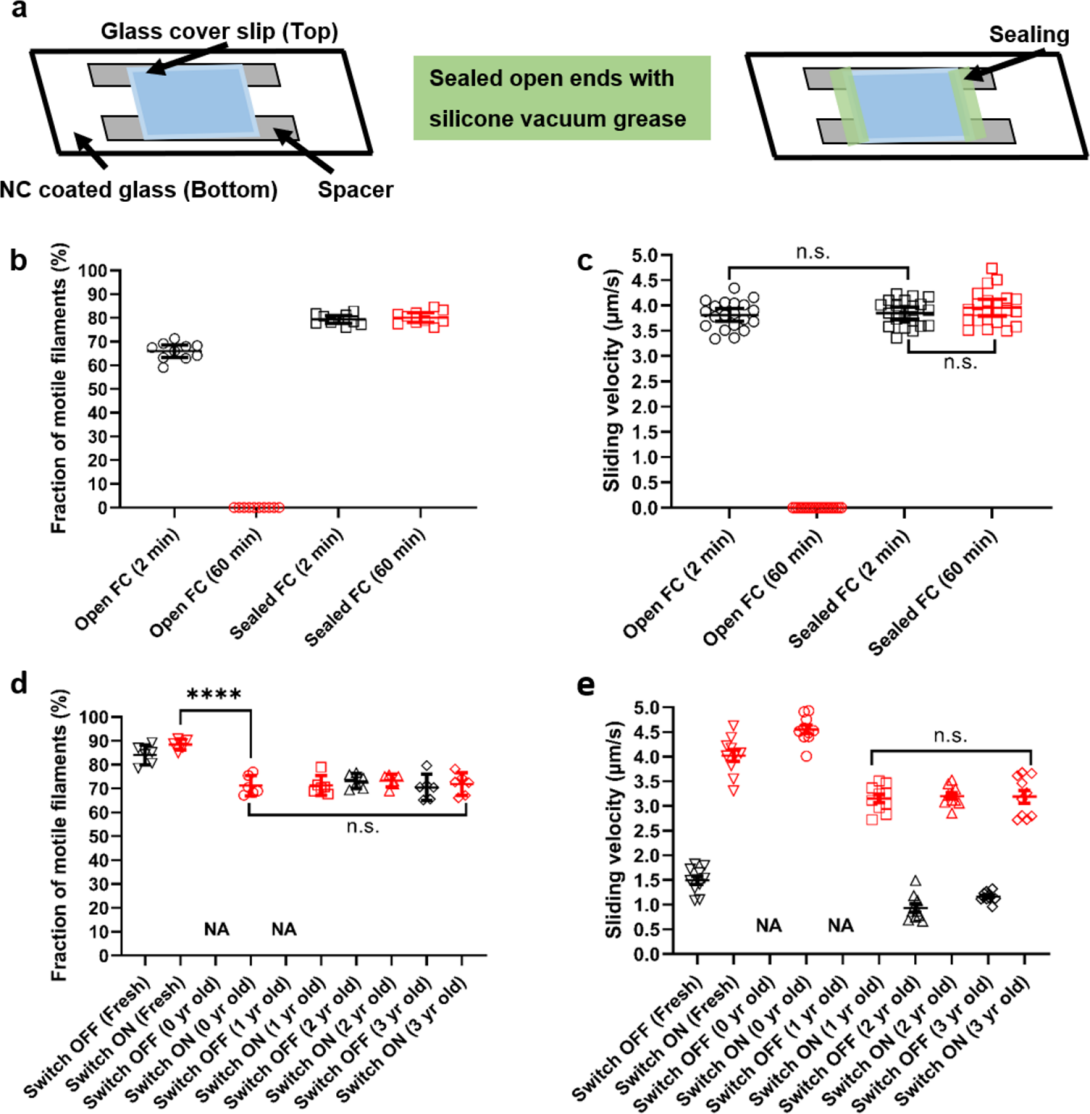
Extended longevity and shelf life of LSM. (a) Schematic of a flow cell used for present experiments, with square coverslip (top), double-sided tape (spacer), and nitrocellulose-coated surface (bottom). Addition of silicon vacuum grease as measure to limit air interaction in the flow cell. (b) Measured fraction of motile filaments with open and sealed flow cells, at 2 and 60 min after addition of assay solution. (c) Measured sliding velocity with open and sealed flow cells (FC), at 2 and 60 min. No statistically significant difference (n.s.) between Open and Sealed FC (2 min after addition of assay solution) (*p* ≈ 0.679). Further, no difference between Sealed FC at 2 and 60 min (*p* ≈ 0.264). (d) Measured fraction of motile filaments after storage of LSM stock solution at −80 °C for different times (year: yr).**** Statistically significant difference, *p* < 0.0001. n.s. No statistically significant difference (*p* ≈ 0.775). (e) Measured sliding velocity after different storage times (cf. (d)). n.s. No statistically significant difference (*p* ≈ 0.928). In (b, c), for fraction, 10 different flow cell regions of interest and for velocity, 20 different filaments were analyzed for each condition distributed with 10 filaments at each of two different experimental occasions (two different days). The slight difference in motile fraction at 2 min may be attributed to variation between different tubes of the same motor stock. Temperature 24.5–25.5 °C. Approximate flow cell size: 18 × 18 mm^2^ and flow cell volume: 20 μL. In (d, e), for fraction, six different flow cell regions of interest and for velocity, 10 different filaments were analyzed for each condition compared from four different year time points. For 0 year and 1-year old stock the data is not available under switched OFF condition. Experiments with 1-, 2-, and 3-year-old stock were performed at temperature 24.5–25.5 °C, motor concentration: 150 nM and antibody dilution ratio: 1/10. The freshly produced stock was tested at Stanford (before transport) at room temperature (23 ± 1 °C), motor concentration: 280 nM and antibody dilution ratio: 1/3. Actin filaments labeled with Rhodamine phalloidin, ON/OFF switching performed using blue LED illuminator (see Materials and Methods), except for case “Fresh” in (d, e) where conditions were as in.^33^ The 0-year-old stock was tested at temperature 23.8 °C, motor concentration: 700 nM and antibody dilution ratio: 1/3. All filaments for a given condition were pooled in the statistical analyses (*t* test in (b, c); *t* test and Analyses of variance [ANOVA] in (d, e). Data shown as mean ±95% confidence intervals superimposed on data for individual filaments.

It is important to optimize the longevity of myosin-propelled actin motility for nanotechnological applications of the motors. In general, with LSM, longevity is less than 60 min after the first addition of the assay solution. We therefore tested whether the method of flow cell sealing, described previously in studies using myosin II motor fragments (HMM),^38^ could prolong motile function. Motility solutions were degassed, and the flow cells (∼18 × 18 mm^2^, roof top surface) were sealed using silicon vacuum grease (Figure 5a). While no motility was observed at 60 min, in the case of open flow cells, motility was maintained in sealed flow cells for more than 60 min, with no significant change in sliding velocity and fraction of motile filaments (Figure 5b,c; *p* > 0.05). Such extension of longevity is beneficial for the future use of light-sensitive motors in applications. Furthermore, the positive effect of this simple intervention lends impression that other previously described procedures for motility due to myosin II motor fragments^38^ may also be beneficial to LSM, possibly extending motile function to several hours.

In addition to the longevity of operation, an extended storage shelf life allows the motors to be used for a long time after their expression and isolation from the cell system. The shelf life has not been systematically investigated previously. It is therefore of interest to note that we here demonstrate only minimal changes in gliding velocity and fraction of motile filaments after storing the motors at −80 °C for more than 3 years (Figure 5d,e), including several days transport of the motors in dry ice from Stanford University, California to Linnaeus University, Sweden. Remarkably, after an initial small decline in fraction of motile filaments with blue light on after the transport (fresh vs 0 year in Figure 5d; *p* < 0.05), this fraction remained constant (*p* ≈ 0.775) over 3 years. Moreover, velocity was constant between year 1 and 3 (p ≈ 0.928). Notwithstanding, the small initial decreases, that may partly be attributed to the transport as well as minor differences in experimental conditions (see legend of Figure 5), the important results in Figure 5 are (i) a maintained function for at least 3 years including similar velocity from year 1 to 3 and fraction of motile filaments from year 0 (after completed transport) to 3, with blue light switched ON and (ii) a maintained switching capability of the motors.

### Toward Selective Function of Motors in Nanochannels

When considering light-switchable motors for the purpose of nanotechnological applications, the generally utilized nitrocellulose substrate is not suitable. Previously, for actin-myosin-based nanotechnological applications, using HMM motor fragments of myosin II, trimethylchlorosilane (TMCS) has instead been utilized to functionalize the surfaces of the nanochannels to make these moderately hydrophobic compared to surrounding hydrophilic polymer resist surfaces.^17,18^ Initially, we tested the adsorption of light-switchable motors directly on TMCS derivatized surfaces without using antibodies, but no motility was observed (Figure S2). Based on the idea that GFP/YFP has a highly negative surface potential at neutral pH^39^ we hypothesized that adsorption of the YFP-motor construct directly on positively charged polylysine surfaces would be possible with maintained motor function. However, repeated tests falsified this idea as no actin motility was observed on polylysine surfaces preincubated with motor constructs at 150 nM. Next, TMCS derivatized glass surfaces and TMCS derivatized silicon dioxide on planar nonpatterned Si wafers were incubated with LSMs after preincubation with anti-GFP antibodies. In both cases, motility was observed (Figure S3), which is of interest because TMCS derivatized surfaces are readily nanopatterned. However, TMCS derivatized planar SiO_2_ surfaces showed better motile function (similar to nitrocellulose) in comparison to the standard TMCS derivatized glass surfaces, demonstrating critically constrained conditions for obtaining selective motility.

**Figure 6:**
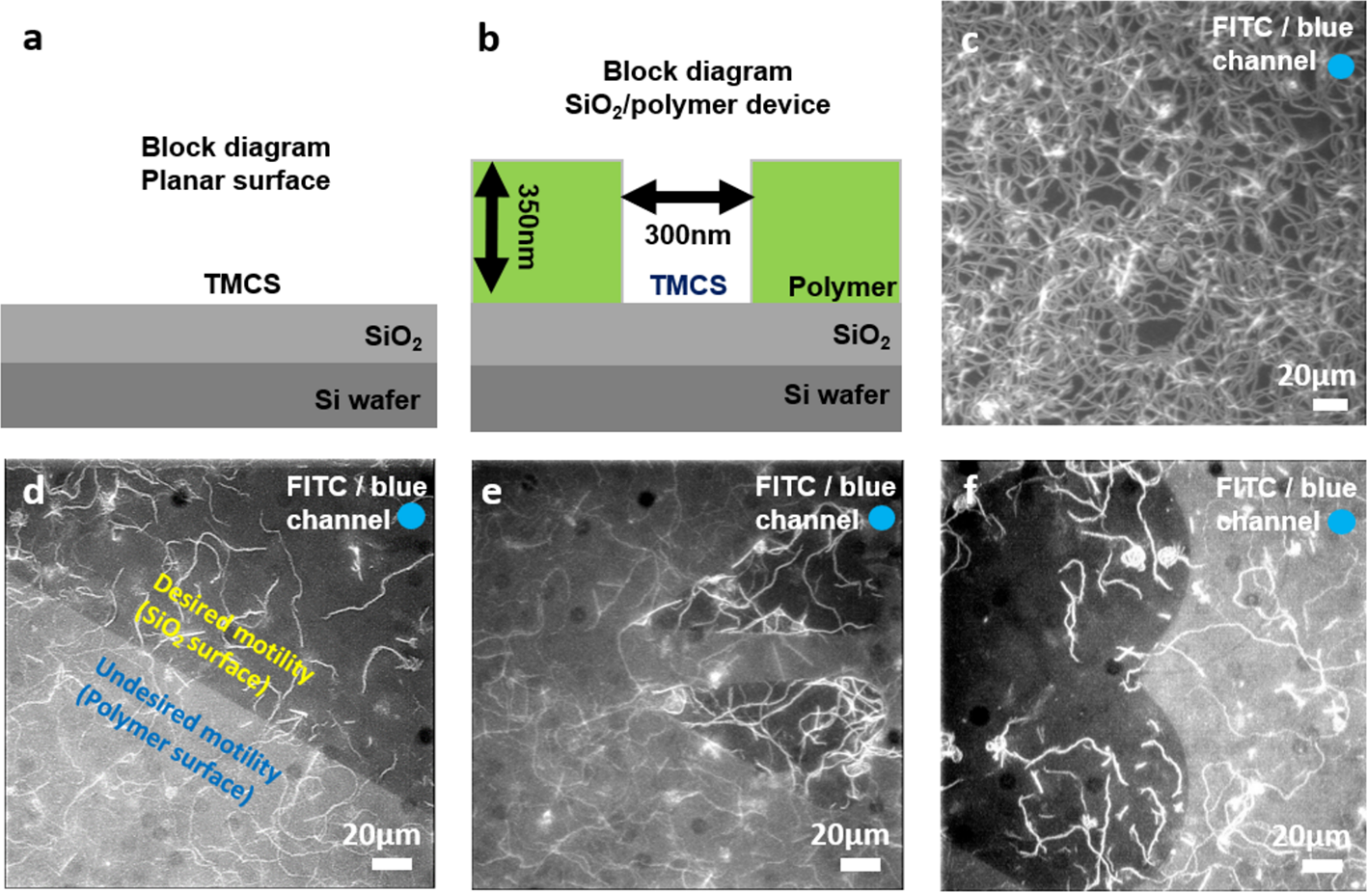
Motility of actin filaments propelled by LSMs on TMCS derivatized planar SiO_2_ and nanofabricated SiO_2_/polymer fabricated devices. (a) Block diagram of a planar SiO_2_ surface. (b) Block diagram of SiO_*2*_/polymer based nanofabricated device. (c) Image stack (100 frames, maximum projection) showing motility on TMCS-derivatized planar SiO_2_surface. (d–f) Image stacks as in b, showing motility on both TMCS-derivatized SiO_*2*_ surfaces (dark) and surrounding polymer (CSAR62; bright). Temperature: 24.5–25.5 °C, Antibody incubation concentration and time: 1:3 and 2 min, LSM incubation concentration: 150 nM. Actin filaments labeled with Alexa-488 phalloidin illuminated with FITC filter set (i.e., LSM motility condition always ON). Blue circle in top right corner indicates color of illuminating light.

To further complicate matters, when motors were tested on TMCS functionalized nanofabricated networks, i.e., TMCS-derivatized SiO_2_ networks with polymer surroundings, motility was observed all over the surface with no selectivity between tracks and surroundings (Figure 6a–f). Similar results were seen whether the polymer CSAR62 or poly(methyl methacrylate) (PMMA) was used. This is in contrast to the selective motility generally observed with the actin myosin II (HMM) system in the nanofabricated networks.^18^ This indicates that low contact angle (<40° on CSAR62^18^ and ∼60 °C on PMMA),^40^ expected for the oxygen plasma treated polymer areas in our nanofabricated network, does not inhibit binding of functional anti-GFP antibodies (used for LSM immobilization).

### Selective LSM Function in Nanochannels

**Figure 7:**
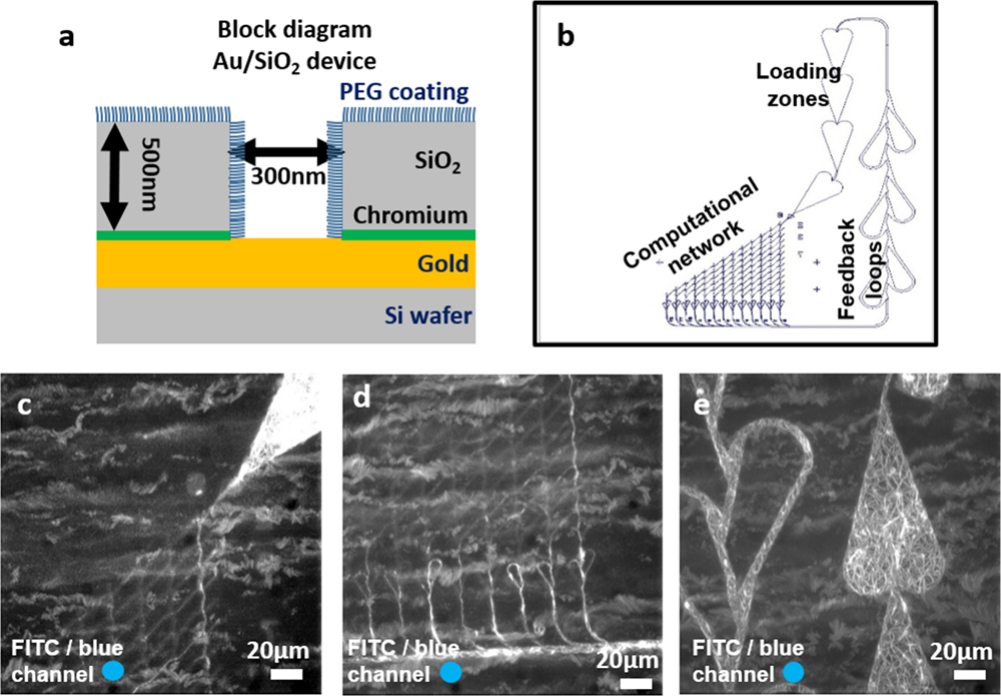
Motility of actin filaments propelled by LSM on Au/SiO_**2**_ fabricated devices coated with PEG-silane. (a) Block diagram of Au/SiO_2_-based nanofabricated device. (b) Schematic of a biocomputation network with labeling of individual regions. (c) Image stack (300 frames, maximum projection) showing motility in the loading zone and completed traveled path in the first nanochannel (from right) of the computational network. (d) Image stack as in (c), showing motility in the first nanochannel (from right) running to the end of the computational network as well as in the nanochannels below rectifier loops at the bottom of the network. (e) Image stack as in (c), showing motility in the loading zones and in the feedback loops (microscale channels). Actin filaments labeled with alexa-488 phalloidin illuminated with FITC filter set (i.e., LSM motility condition always ON). Blue circle in the bottom left corner indicates the color of illuminating light.

With the observed poor selectivity of motility on SiO_2_/polymer-based nanofabricated networks of the types conventionally used for nanodevices for myosin II motor fragments (see above), other material combinations were tested. Previously, Au/SiO_2_-based nanofabricated devices have been used for the kinesin-1/microtubule motor system^41^ (Figure 7a). Interestingly, we observed selective motile function on such nanofabricated networks designed for network-based biocomputation (Figure 7b–e). However, in the biocomputation network paths tested, motility was not observed in all the valid paths (Figure 7c,d; Movie S2). These problems require network optimization with regards to details in the surface chemistry derivatization procedure and, possibly, the nanochannel geometry, such as the channel depth and width. The idea of problems with the surface functionalization and its homogeneity is consistent with subsequent experiments using different batches of Au/SiO_2_–PEG (PEG = poly(ethylene glycol)) biocomputation networks or other networks with nanochannels. In these studies, motility was not observed in a consistent and uniform manner (Figure S4). If motility was observed at all, it was seen mainly in the relatively large micrometer-scale regions but without motility in the nanochannels. To circumvent such issues and achieve reproducible fabrication of devices, systematic and extensive investigations will be required, including both the nanofabrication, surface functionalization, storage, and chip transportation processes. Possibly, a major challenge in achieving reproducibility with actomyosin is the requirement to use nanochannels (100–200 nm wide) with gold-coated surfaces rather than microchannels (∼1 μm wide), which was feasible in previous work^41^ using the microtubule-kinesin motor system. Nonetheless, what is important is that we have demonstrated that the type of gold-based nanochannel system in Figure 7 is highly likely to be a viable approach once it has been optimized in all details.

**Figure 8:**
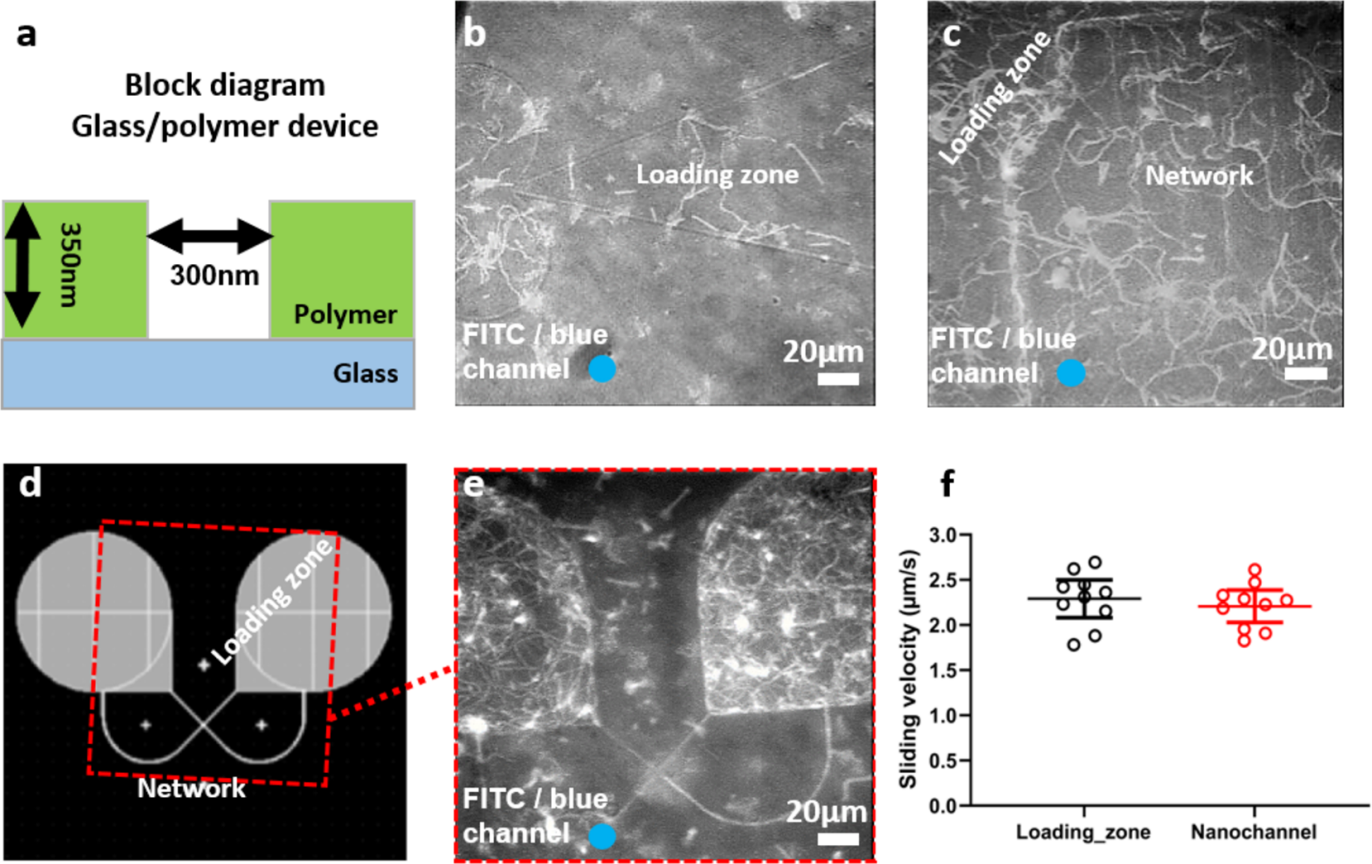
Motility of actin filaments produced by LSMs on glass/polymer fabricated devices with and without treatment with 1% pluronic F-127. (a) Block diagram of glass/polymer-based nanofabricated device. (b) Image stack (300 frames, maximum projection) showing nanodevice with low motility in the loading zone and no attachment in the surrounding polymer region, when treated with 1% pluronic F-127. (c) Image stack as in (b), showing fabricated device with good motility all over the network, that is, on loading zone, nanochannels, and also on the surrounding polymer region, without treatment with 1% pluronic F-127. (d) Schematic of a loop design for a glass/polymer fabricated device. (e) Image stack as in (b), showing test loop fabricated device (as in (d)) with motility both in the loading zones and in the nanochannels, without treatment with 1% pluronic F-127 (see also Movie S3). (f) Measured sliding velocity both in the loading zone and nanochannels, analyzed from the experimental occasion shown in (e). Data are given as mean ±95% confidence intervals superimposed on data for individual filaments. Temperature, 24.5–25.5 °C. Actin filaments labeled with Alexa-488 phalloidin illuminated with FITC filter set (i.e., LSM motility condition always ON). Blue circle in bottom left corner indicates color of illuminating light.

With the observed inconsistency of motile function in Au/SiO_2_–PEG-based nanofabricated devices (Figure S4), another possibility was explored with nanochannel floors made up of glass and the surroundings made up of polymer (PMMA) (Figure 8a). For the purpose of selective motility, nanofabricated devices were incubated for 2 h with 1% pluronic F-127 before being incubated with antibodies and light-sensitive motors. When *in vitro* motility assay studies were performed using such surfaces, low actin filament attachment and motility was observed in nanochannels in the network region (Figure 8b; Movie S3). On the other hand, good motility was observed across the network (both on glass and PMMA including nanochannels) if the treatment with pluronic F-127 was omitted (Figure 8c–e). Similar motility across the chip was also observed previously when SiO_2_/polymer nanofabricated devices were used (Figure 5e). On another occasion when we excluded pluronic F-127 treatment, good motility, similar to that observed in the loading zone region, was achieved in the nanochannels (∼300 nm width) for loop design fabricated devices (Figure 8d–f). Importantly, the studies suggest that the nonfunctionalized glass surfaces provide useful substrates for adsorption of antibodies in a conformation that can bind engineered motors with YFP and support motile function of the light-switchable motors. Furthermore, the studies show that pluronic has the capacity to block motility. Thus, an alternative viable strategy to achieve selective motility only in nanochannels could be the incubation with a suitable concentration of pluronic of a nanostructured surface that combines nanochannels with hydrophilic SiO_2_ floors and a hydrophobic polymer for the surrounding of the channels. Unfortunately, however, despite testing a range of different pluronic incubation concentrations and incubation times in the range 0.001%–1% and 1 min–2 h we did not achieve selective function in the nanochannels after subsequent incubation with anti-GFP antibodies, LSM, actin filaments, and assay solution. In order to pursue this strategy, it will presumably be necessary to fine-tune the difference in hydrophobicity between the nanochannel surfaces and the surroundings by modifications of the nanofabrication procedure. Experiments using nitrocellulose-coated surfaces (Figure S5) suggest that it should be possible to achieve selectivity with motility only in the nanochannels by lower pluronic adsorption compared to the surroundings. Thus, in these experiments (Figure S5), a stepwise increase in incubation time from 1 to 3 min using a pluronic F-127 working concentration of 0.01% led to stepwise reduction in the sliding velocity and motility distribution following subsequent addition of anti-GFP antibodies, LSM, actin filaments. and assay solution.

Above we describe two possible approaches to achieve selective motility using LSM in nanofabricated networks, either using channels with gold floors surrounded with PEG-coated SiO_2_ or SiO_2_ floors surrounded with pluronic-coated hydrophobic polymer. Our repeated attempts to consistently achieve such nanonetworks met appreciable challenges, despite efforts to also modify the procedures. However, we also clearly demonstrate that both approaches are, in principle, feasible but a range of time-consuming optimizations will be required before consistent application.

## Conclusions

In conclusion, the results provide groundwork for the integration of light-switchable motors into nanofabricated devices. Improvements have been made to obtain suitable motor density, dependent on the antibody density on the surfaces. Other developments for the use of LSM in nanodevices is our demonstration of several years of shelf life at −80 °C and prolongation of motile function by implementing methods of solution degassing and flow cell sealing. This suggests that several other approaches previously developed for myosin II motor fragments may expand longevity further.^38^ We further studied the feasibility of motility in nanochannels with lack of motility in surrounding areas, testing different surface chemistry combinations: 1) TMCS-derivatized SiO_2_/polymer, 2) Au/SiO_2_ treated with PEG-silane, and 3) glass/polymer nanostructures treated with pluronic-F-127. With Au/SiO_2_-based nanodevices, selective and good motile function was achieved in one experiment, providing sufficient information that use of light-switchable motors would be feasible for sorting applications or for programming of network-based biocomputation devices. However, the reproducibility of Au/SiO_2_–PEG-based nanodevices was not satisfactory, and for the nanodevices with TMCS-SiO_2_/polymer and glass/polymer-pluronic, further optimizations are needed for selective functionality. Our results also suggest that effective switching would benefit from engineering of the motors to robustly achieve even higher motility contrast between “ON” and “OFF” conditions (Figure 4) while still retaining high gliding velocities in comparison to previous light-switchable motors based on myosin VI.^32^

## Materials and Methods

### Materials

Anti-Green Fluorescent protein (GFP) antibody was purchased from Millipore (catalog no. MAB3580), following previous studies that used this antibody for surface attachment of YFP-tagged myosin motors.^33,43^ The SF9 cell lines were purchased from Invitrogen (catalog no. 11496-01). Serum-free cell medium (sf-900TM II SFM 1x) was purchased from Life Technologies (catalog no. 10902-096). Glass coverslips were purchased from VWR (22 mm × 50 mm, #1.5, catalog no.16004-336) and Histolab (24 mm × 60 mm, #0, catalog no. 6772). Nitrocellulose was purchased from Ladd research and Sigma-Aldrich. Rhodamine Phalloidin, Alexa-488 Phalloidin, and Alexa-647 Phalloidin were obtained from Thermo Fisher Scientific. Adenosine triphosphate (ATP) was purchased from either Sigma-Aldrich or from Calbiochem. MgCl_2_ is from Fisher Bioreagents. Tris was purchased from Thermo Scientific. All other biochemical reagents were of analytical or biotechnological grade and purchased from Sigma-Aldrich unless otherwise stated.

### Buffers and Solutions

Tris assay buffer (Buffer A; pH 8.0) was composed of 25 mM Tris, 25 mM KCl, 10 mM dithiothreitol (DTT), 2 mM MgCl_2_, 1 mM potassium ethylene glycol-bis(β-aminoethyl ether)-*N*,*N*,*N*,*N*-tetraacetic acid (K_2_EGTA). Wash buffer (Buffer B; pH 8.0) containing bovine serum albumin (BSA) was prepared by adding 2 mg/mL BSA in Buffer A. Assay solution (Buffer C; pH 8.0) for gliding *in vitro* motility assays was prepared in Buffer A with 0.4% glucose, 25 U/ml glucose oxidase, 2000 U/ml catalase, 1 mM creatine phosphate, 0.001 mg/mL creatine phosphokinase, and 2 mM MgATP. Myosin and actin were diluted in Buffer A.

In some of the experiments, we also used a low-ionic strength solution (LISS) as an alternative to the Tris assay buffer. The LISS solution was prepared with a mixture of the following components: 1 mM magnesium chloride (MgCl_2_), 10 mM 3-(*N*-morpholino)propanesulfonic acid (MOPS), and 0.1 mM potassium ethylene glycol-bis(β-aminoethyl ether)-*N*,*N*,*N*,*N*-tetraacetic acid (K_2_EGTA). The ionic strength of the LISS solution was 15 mM, and pH was set to 7.4. Using LISS as the base buffer, wash buffer and assay buffer were prepared as described in the following. For the wash buffer, LISS was supplemented with 1 mM DTT and 50 mM KCl (final concentrations). For assay buffer, the final ionic strength was 60 mM, where LISS was supplemented with 45 mM KCl, 10 mM DTT, 1 mM magnesium adenosine triphosphate (MgATP), 3 mg mL^–1^ glucose, 2.5 mM creatine phosphate (CP), 0.2 mg mL^–1^ creatine phosphokinase (CPK), and oxygen scavenger mixture (GOC): 0.1 mg mL^–1^ glucose oxidase and 0.02 mg mL^–1^ catalase.

### Protein Preparations

The engineered myosin construct: MyLOVChar4^33^ was expressed in SF9 cell lines by transient transfection and then purified following a published protocol.^44^ The MyLOVChar4 construct includes codons for a C-terminal yellow fluorescent protein, flexible linker, and a FLAG tag (DYKDDDDK). Purified proteins were characterized by Sodium Dodecyl Sulfate-Poly Acrylamide Gel Electrophoresis (SDS-PAGE), and concentration was determined by calculating band intensity using ImageJ.^45^ Purified myosins were snap-frozen with liquid N_2_ on the day of protein purification and stored at −80 °C until use. Actin was prepared from rabbit fast skeletal muscle as described earlier.^2,46^ Actin filaments were labeled with either Rhodamine Phalloidin or Alexa-488 Phalloidin or Alexa-647 Phalloidin and stored at 4 °C until use.

### Surface Preparations

Nitrocellulose coating of glass coverslips (Figure 2) was performed either by spin coating (0.1% nitrocellulose in amylacetate) or by spreading with a pipet (1% nitrocellulose) tip before assembling into a flow chamber. Surface derivatization with trimethylchlorosilane (TMCS) on glass coverslip (Figures S2 and S3) surfaces was carried out as described previously.^34,35^

The patterned surfaces in Figure 6 were made on a 2 in. Si(100) wafer with a 70 nm thick SiO_2_ layer deposited by atomic layer deposition. The wafer was cleaned in an ultrasonic bath in acetone (VWR, Radnor, PA, USA) and isopropanol (VWR, Radnor, PA, USA) for 3 min each. The wafer was spin-coated with a layer of polymer resist CSAR62 (Allresist, Strausberg, Germany) or PMMA dissolved in anisole (VWR, Radnor, PA, USA) to 13% at 5000 rpm for 30 s and baked on a hot plate for 2 min at 180 °C. The resist was patterned by electron beam lithography (Voyager, Raith GmbH, Dortmund GmbH) at 50 kV acceleration voltage, beam current ∼0.57 nA, and 250 μC/cm^2^ dose and developed in amyl acetate (Sigma-Aldrich, Saint Louis, MO, USA) for 90 s while stirring. This was followed by rinsing in isopropanol and drying under nitrogen flow. After development, the wafer was diced into 10 × 10 mm^2^ samples and treated with oxygen plasma for 15 s at 5 mbar. The unpatterned surfaces were made on 10 × 10 mm^2^ Si(100) samples with a 70 nm layer of SiO_2_ deposited by atomic layer deposition. Both the patterned and unpatterned samples were silanized in trimethylchlorsilane (TMCS) for 64 min at 200 mbar, as previously described.^18^ Planar SiO_2_ surfaces were made following the same fabrication method except that the sample was plasma-ashed in “Plasmapreen” for 30 s at 5 mbar and the silanization in TMCS was for 35 min at 200 mbar.

For some of the experiments, different versions of the nanofabricated biocomputational and other devices were used. These nanodevices consisted of Au floor, SiO_2_ walls, and Cr layer to support adhesion between Au and SiO_2_ (Figures 7 and S4). These chips were nanofabricated using electron beam lithography as described elsewhere.^15,41^ Briefly, then, the developed chips were cleaned in acetone for 10 min, followed by rinsing with ethanol and distilled water. Next, devices were treated with poly(ethyleneoxy)-silane (PEG-silane) (2.4 mg/mL) for at least 16 h (PEG-silane; 90%; ABCR, SIM4492.7) dissolved in toluene-HCl. After this, devices were rinsed further in toluene, ethanol, and distilled water. This PEGylation was performed to obtain selective prevention of the motor protein binding on the SiO_2_ walls and other areas surrounding the motor tracks. Other types of nanodevices were nanofabricated using a glass floor and PMMA walls (Figure 8), with additional treatment with 1% pluronic F127.

### In Vitro Motility Assays and Recording

Flow cells for the gliding filament assay were assembled with the motility supporting surface or chip (*e.g*., nitrocellulose-coated glass, trimethylchlorosilane derivatized glass/SiO_2_, and nanofabricated chip) appropriately oriented to be compatible with the microscopy setup and an untreated glass coverslip for the other surface with the two surfaces separated by spacers in the form of double-sided tapes. The flow cell was first incubated with Anti-Green Fluorescent Protein Antibody (anti GFP antibody). Incubation time was 2 min for nitrocellulose-coated surfaces at room temperature. Next, the surface was blocked with a BSA-containing buffer (buffer B) followed by myosin incubation for 2 min. Then, the flow cell was rinsed with the buffer B followed by incubation with fluorescently labeled actin filaments (using rhodamine Phalloidin, Alexa-488 Phalloidin, or Alexa647 Phalloidin) for 2 min. Finally, assay solution (Buffer C) was added before recording the myosin-induced actin sliding movement.

Image acquisition was performed by using an inverted fluorescence microscope (Zeiss Axio Observer.D1). A 532 nm optically pumped semiconductor laser (Coherent) or a mercury short-arc lamp (OSRAM GmbH) was used together with suitable filter sets allowing observation of rhodamine, Alexa-488, or Alexa-647 fluorescence. Image sequences were recorded using an electron multiplying charge-coupled device (EMCCD) camera (C9100-12PHX1, Hamamatsu Photonics; or ANDOR iXON^EM^+; model DU-897E-CS0-#BV). Images were recorded using a frame rate in the range of 4–10 frames/second. Actin filament gliding velocities were calculated as described earlier.^20,47^

### Illumination Conditions for Motility ON and OFF

Illumination in the microscope was obtained using the 100 W HBO Mercury short-arc lamp (OSRAM GmbH) through a discrete Zeiss FL attenuator (catalog no. 423647, Zeiss) at position 5 (transmission ∼20%) corresponding to an irradiance of 1.5 W/cm^2^. Fluorescence filter sets were selected based on the labeling dye of the actin filaments: FITC fluorescence filter set (excitation bandpass 450–490 nm) for Alexa-488 Phalloidin, Cy3 fluorescence filter set (excitation bandpass 537–563 nm) for Rhodamine Phalloidin, Cy5 fluorescence filter set (excitation bandpass 625–655 nm) and/FITC/Cy5 dual band H fluorescence filter set (excitation bandpasses 450–490 nm and 601–645 nm, AHF, Germany) for Alexa-647 Phalloidin.

When the actin filaments were labeled with Rhodamine Phalloidin, ON motility conditions were obtained using blue light illumination from a light-emitting diode (LED) source as described previously.^32,33^ Briefly, blue light illumination was achieved using a light-emitting diode (LED) source (M470L3, middle wavelength of 470 nm, Thorlabs). The source was placed 4–8 cm above the flow channel with an intensity value of 4 in the LED controlling knob. Here, the microscope illumination through the Cy3 filter set was always active during both ON and OFF states of the LSM motility conditions to constantly observe the actin filaments.

Light irradiance of the LED source positioned 4–8 cm above the sample was estimated using USB Power and Energy Meter device (PM100USB, ThorLabs) equipped by Silicon Power Head light sensor (400–1100 nm, 50 mW, ThorLabs), controlled by Optical Power Monitor (v. 4.1) software provided by the manufacturer. Positioning the LED source 4–8 cm above the light sensor created a beam diameter that was larger than the sensor (9.5 mm). Thus, the light irradiance could be directly measured by the device, assuming a Gaussian beam profile. All measurements consisted of 10 s recordings with at least 3000 readings (n) from which mean value and corresponding standard deviation was calculated. This resulted in light irradiance of 4.987 ± 0.002 mW/cm^2^ (4 cm above sensor) and 1.326 ± 0.001 mW/cm^2^ (8 cm above sensor). Importantly, we found no difference in ON/OFF switching behavior depending on the illumination system used.

In experiments when the actin filaments were labeled with Alexa-488 Phalloidin, ON motility conditions were obtained directly from the microscope lamp source using the FITC filter cube allowing observation of the filaments only in ON conditions. In cases where the filaments were labeled with Alexa-647 Phalloidin, the dual transmission FITC/Cy5 filter cube was used, allowing observation of the filaments both in ON and OFF conditions. To estimate the light irradiance through FITC filter used to obtain the ON state for the LSM-driven motility, we first measured the total light intensity at the sample plane. The light sensor was placed on top of a glass coverslip, which was in contact with the objective via immersion oil, effectively mimicking the real experimental setup. Before the power reading, the microscope field stop was closed until the field stop image on the sample plane is just outside the field of view in the eyepiece.^48^ To properly adjust the objective focus to the sample plane and the field stop opening, 5 μL of fluorescent beads or highly concentrated (100 nM) Alexa-488 Phalloidin labeled actin filaments was placed between two glass coverslips. Under such conditions, the light power measurement resulted in 1.65 ± 0.01 mW (mean ± standard deviation (SD)). An irradiance of 1.5 W/cm^2^ was then calculated by dividing the measured power with an area of the field of view (1.0752 × 10^–3^ cm^2^) observed through the eyepiece. The irradiance of blue light was not directly measured for the case where we observed Alexa-647 Phalloidin labeled filaments with dual filter FITC/Cy5, as the sensor would detect both FITC and Cy5 excitation light intensities. However, since the excitation bandpass 450–490 nm for blue light is the same in FITC and in dual FITC/CY5 filters, the blue light irradiances are also most likely very similar (i.e., 1.5 W/cm^2^).

### Statistical Analysis

In similarity to previous experiments using HMM from fast skeletal muscle to propel actin filaments^49−51^ each filament is treated as an independent random sample (n: sample size) from one given population in experiments on different occasions. This assumption is supported by the present data. Thus, in Figures 4h and 5c the velocity data overlapped and showed no significant differences in mean values (*p* > 0.05) between two experimental occasions and between two different flow cells on the same occasion (Figure 5c). The same applied for the velocity data in Figure 5e (between years 1, 2 and 3), neglecting the differences between these data and the earlier data where the experimental conditions differed. The evidence for the fraction of motile filaments at each area of a flow cell surface as independent random samples is somewhat less convincing. Thus, there is a small variability between flow cells in Figures 4g and 5b but not in Figure 5d (years 0–3) suggesting that the assumption of independence and sampling from one given population is only approximately valid. Consequently, we consider statistical analyses of changes in fraction of motile filaments as strictly valid only for changes observed with time in a given flow cell. In contrast we confidently pool data between experimental occasions for statistical analyses of changes in velocity. Finally, based on the scatter plots of the data and generally *n* > 20, we assume Gaussian distributions of the populations leading us to analyze the data using two-sided Student’s *t* test and one-way Analyis of Variance (ANOVA) for single and multiple group comparisons, respectively. The statistical analyses were performed using Graph Pad Prism v 9.3.1. When ANOVA was used, this was followed by a post hoc test for linear trend in Figure S4. Statistical significance is concluded if *p* < 0.05. Data are shown as mean ±95% confidence interval unless otherwise stated.

## Supporting Information Available

Movie (S1): Swithching of LSM driven motility between low motility state (“OFF”) and high motility state (“ON”) (AVI)
Movie (S2): Motility under blue light in gold nanochannels surrounded by PEG coated SiO_2_ (AVI)
Movie (S3): Motility under blue light in nanochannels with glass floor surrounded PMMA polymer without Pluronics treatment (AVI)
Figures: (S1) Proposed practical implementation of idea for programmable computation in main Figure 1. (S2) lack of motility of actin filaments driven by light switchable motors (LSMs) attached directly on TMCS-derivatized glass surface, (S3) motility of actin filaments with blue light, produced by LSMs immobilized to different surfaces via anti-GFP antibodies, (S4) motility function of LSMs in three different experiments on Au/SiO_2_ chips coated with PEG-silane with inconsistent outputs and (S5) motility of actin filaments produced by LSMs on nitrocellulose coated glass with treatment of 0.01% pluronic-F127 for three different time periods (PDF)

## Supplementary Material

nn3c05137_si_001.avi

nn3c05137_si_002.avi

nn3c05137_si_003.avi
