# Supplementary material for: Exploitation of Engineered Light-Switchable Myosin XI for Nanotechnological Applications
Source: ACS Nano. 2023 Aug 28;17(17):17233–44. doi: 10.1021/acsnano.3c05137 (PMC10510702; doi:10.1021/acsnano.3c05137)
Supplement: Supplementary file 4 — nn3c05137_si_004.pdf [file nn3c05137_si_004.pdf]

Supporting Information

Exploitation of engineered light-switchable myosin XI for nanotechnological applications

Aseem Salhotra[1,3,*], Mohammad Ashikur Rahman[1*], Paul Ruijgrok[2], Christoph Robert Meinecke[4], Marko Ušaj[1], Sasha Zemsky[2], Frida W Lindberg[3], Roman Lyttleton[3], Heiner Linke[3], Till Korten[5], Zev Bryant[2], and Alf Månsson[1a)]

[1]Department of Chemistry and Biomedical Sciences, Linnaeus University, Sweden.
[2]Department of Bioengineering, Stanford University, Stanford, California 94305, USA.
[3]NanoLund and Solid State Physics, Lund University, Box 118, 22100 Lund, Sweden.
[4]Center for Microtechnologies, Technische Universität Chemnitz, Chemnitz, 09126, Germany.
[5]B CUBE - Center for Molecular Bioengineering and Physics of Life, Technische Universität Dresden, D-01307 Dresden, Germany.

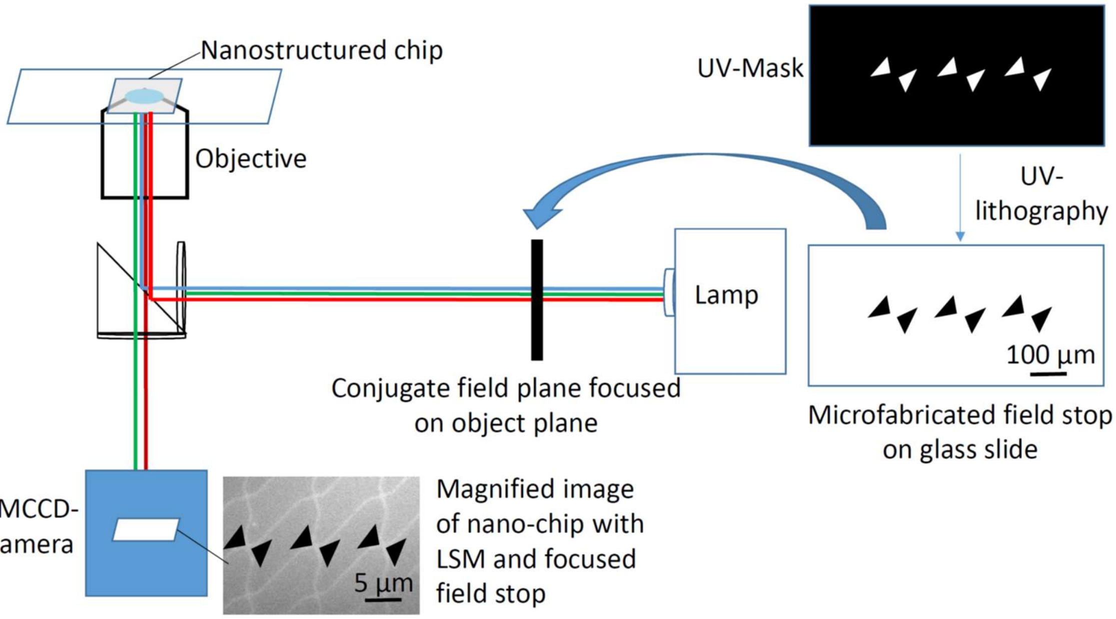

Figure S1. Proposed practical implementation of idea for programmable computation in main Figure 1.

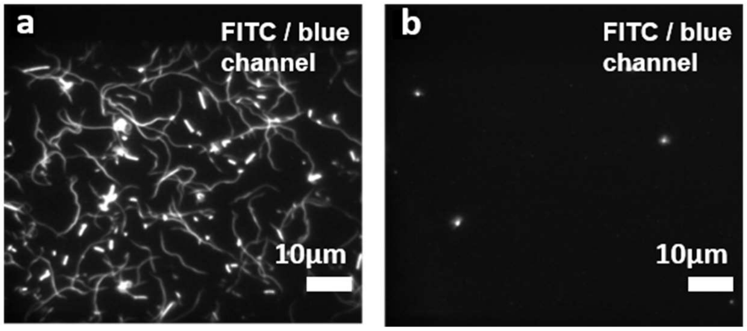

Figure S2. No motility of actin filaments driven by light switchable motors (LSMs) attached directly on TMCS-derivatized glass surface. *a) Image stack (100 frames, maximum projection) on a standard nitrocellulose coated glass, motors attached to the surface via anti-GFP antibodies. b) Image stack as in a, on TMCS-derivatized glass. Motors adsorbed directly to the surface without pre-addition of antibodies. Illumination using FITC filter set in fluorescence microscope. Actin filaments labelled with Alexa-488 phalloidin. Note, very few actin filaments on TMCS derivatized glass in b and no motility.*

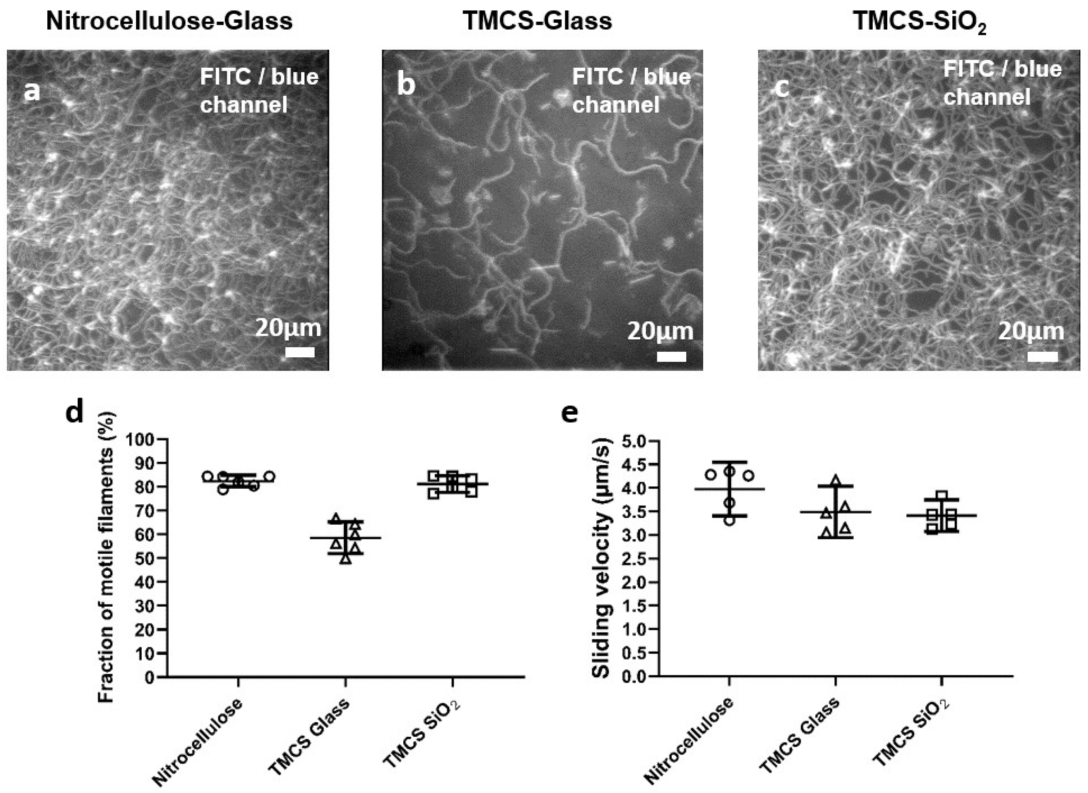

Figure S3: Motility of actin filaments with blue light, produced by LSMs immobilized to different surfaces via anti-GFP antibodies. *a) Image stack (100 frames, maximum projection), showing uniform distribution of motility across the nitrocellulose coated glass surface. b) Image stack as in a, showing comparatively lower distribution of motility across the TMCS-derivatized glass surface. c) Image stack as in a, showing high distribution of motility across the TMCS-derivatized* SiO_2 *on an Si wafer. d) Fraction of motile filaments on nitrocellulose, TMCS-glass and TMCS-*SiO_2*. e) Sliding velocity on nitrocellulose, TMCS-glass and TMCS-*SiO_2*. In d) and e), for the fraction of motile filaments, six different flow cell regions of interest were observed. For velocity, five different filaments were analysed for each condition distributed over two different experimental occasions. Temperature 24.5 °C – 25.5 °C. Illumination using FITC filter set in fluorescence microscope. Actin filaments labelled with Alexa-488 phalloidin. Data shown as mean ± 95 % confidence intervals superimposed on data for individual filaments.*

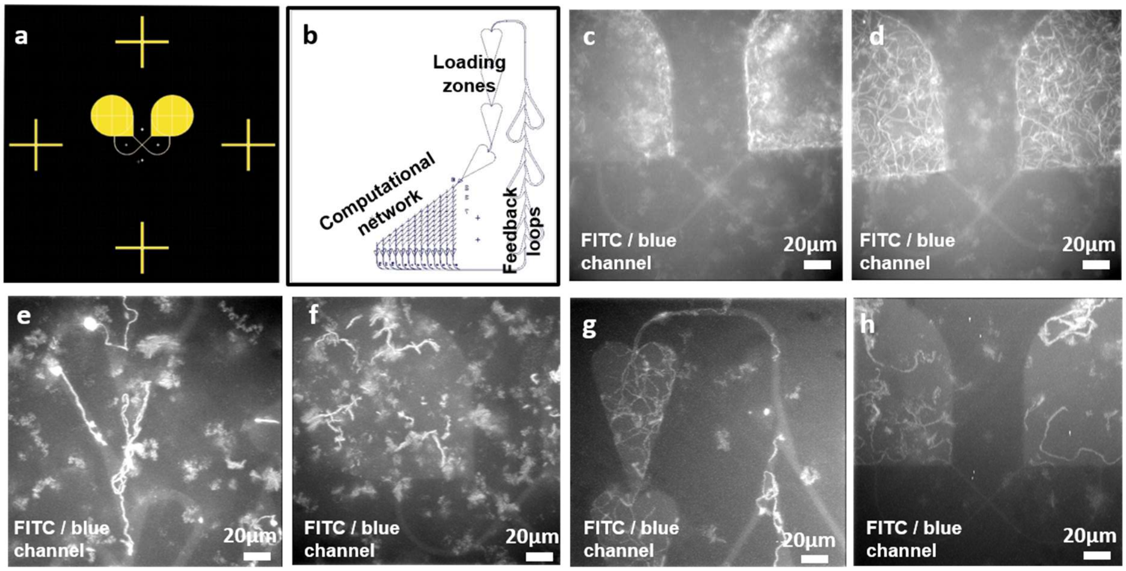

Figure S4. Motility function of LSMs in three different experiments on Au/SiO_2 chips coated with PEG-silane with inconsistent outputs. *a) Schematic of a test network with looped nano channels. b) Schematic of a biocomputation network (Image is a repeat of Figure 7.b). c) Image stack (300 frames, maximum projection) showing loading zones with partial motility and nano channel without motility (Experimental occasion 1). d) Image stack (300 frames, maximum projection) showing loading zones with evenly distributed motility and nano channel without motility (Experiment occasion, 1). e) Image stack (200 frames, maximum projection) showing feedback loop with low partial motility (Experimental occasion 2). f) Image stack (200 frames, maximum projection) showing loading zones with low partial motility and nano channels without motility (Experimental occasion, 2). g) Image stack (300 frames, maximum projection) showing loading zones and feedback loop with low partial motility (Experimental occasion 3). h) Image stack (300 frames, maximum projection) showing loading zones with low partial motility and nano channels without motility (Experimental occasion, 3). Illumination using FITC filter set in fluorescence microscope. Actin filaments labelled with Alexa-488 phalloidin.*

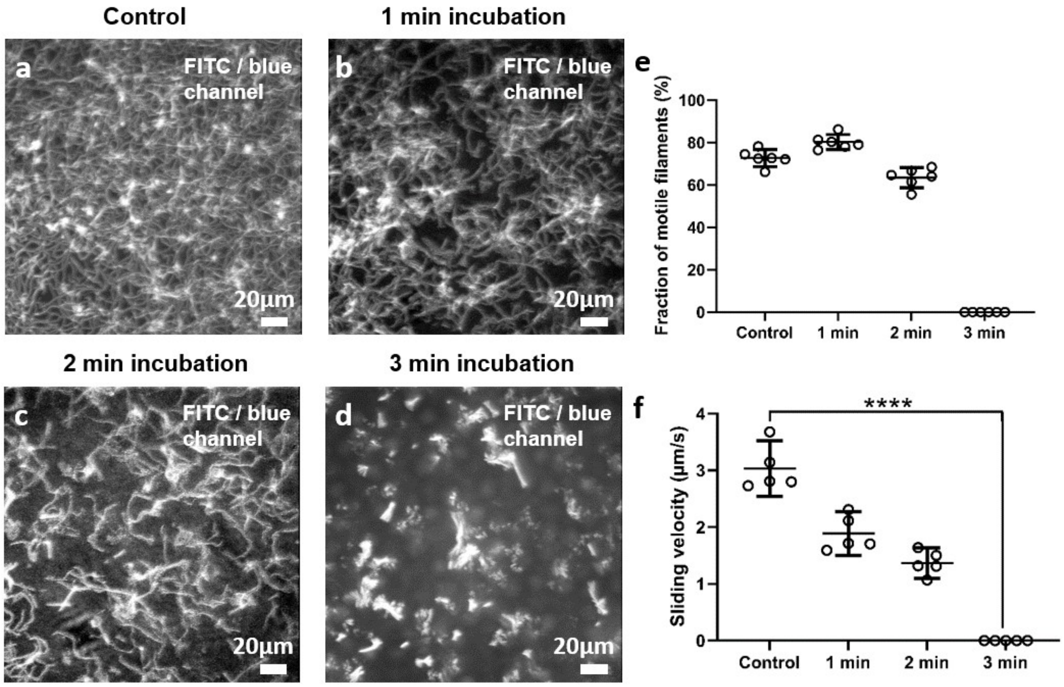

Figure S5. Motility of actin filaments produced by LSMs on nitrocellulose coated glass with treatment of 0.01% pluronic-F127 for three different time periods. *a) Image stack (100 frames, maximum projection) showing dense distribution of motility in control, without any pluronic-F-127 treatment. b) Image stack as in a, showing slightly lower distribution of motility compared to control, when treated with 0.01% pluronic-F-127 for 1 min. c) Image stack as in a, showing sparse motility compared to control, when treated with 0.01% pluronic-F-127 for 2 min. d) Image stack as in a, showing no motility, when treated with 0.01% pluronic-F-127 for 3 min. e) Fraction of motile filaments for control, 1, 2, and 3 min incubation of 0.01% pluronic-F-127. f) Sliding velocity for control, 1, 2, and 3 min incubation of 0.01% pluronic-F-127. In e) and f), for fraction of motile filaments, six different flow cell regions of interest were evaluated. For velocity, five different filaments were analysed for each condition distributed over two different experimental occasions. Temperature 24.5 °C – 25.5 °C. Illumination using FITC filter set in fluorescence microscope. Actin filaments labelled with Alexa-488 phalloidin. Data shown as mean ± 95 % confidence intervals superimposed on data for individual filaments. Statistical analysis in panel f by one way ANOVA showing statistically significant difference (**** p<0.0001) followed by post-hoc analysis for trend (p<0.0001).*

Supporting Movie legends

Movie S1. Swithching of LSM driven motility between low motility state (off) and high motility state (on). Actin filaments labelled with Rhodamine phalloidin. Switching performed using top blue diode illuminator (see Materials and Methods). Movie accelerated to double real frame rate.

Movie S2. Motility under blue light in gold nanochannels surrounded by PEG coated SiO_2. Actin filaments labelled with Alexa-488 phalloidin illuminated with FITC filter set of fluorescence microscope. Movie accelerated to double real frame rate.

Movie S3. Motility under blue light in nanochannels with glass floor surrounded PMMA polymer without Pluronics treatment. Actin filaments labelled with Alexa-488 phalloidin illuminated with FITC filter set of fluorescence microscope as in Movie S2. Note motility, albeit of rather low quality, particularly on glass surfaces. Movie accelerated to double real frame rate.
